# Association between long-term exposure to air pollution and immune-mediated diseases: a population-based cohort study

**DOI:** 10.1136/rmdopen-2021-002055

**Published:** 2022-02-15

**Authors:** Giovanni Adami, Marco Pontalti, Giorgio Cattani, Maurizio Rossini, Ombretta Viapiana, Giovanni Orsolini, Camilla Benini, Eugenia Bertoldo, Elena Fracassi, Davide Gatti, Angelo Fassio

**Affiliations:** 1 Rheumatology Unit, Department of Medicine, University of Verona, Verona, Italy; 2 Italian Institute for Environmental Protection and Research, Rome, Italy

**Keywords:** autoimmunity, arthritis, autoimmune diseases, outcome assessment, health care

## Abstract

**Objective:**

Environmental air pollution has been associated with disruption of the immune system at a molecular level. The primary aim of the present study was to describe the association between long-term exposure to air pollution and risk of developing immune-mediated conditions.

**Methods:**

We conducted a retrospective observational study on a nationwide dataset of women and men. Diagnoses of various immune-mediated diseases (IMIDs) were retrieved. Data on the monitoring of particulate matter (PM)10 and PM2.5 concentrations were retrieved from the Italian Institute of Environmental Protection and Research. Generalised linear models were employed to determine the relationship between autoimmune diseases prevalence and PM.

**Results:**

81 363 subjects were included in the study. We found a positive association between PM10 and the risk of autoimmune diseases (ρ+0.007, p 0.014). Every 10 µg/m^3^ increase in PM10 concentration was associated with an incremental 7% risk of having autoimmune disease. Exposure to PM10 above 30 µg/m3 and PM2.5 above 20 µg/m^3^ was associated with a 12% and 13% higher risk of autoimmune disease, respectively (adjusted OR (aOR) 1.12, 95% CI 1.05 to 1.20, and aOR 1.13, 95% CI 1.06 to 1.20). Exposure to PM10 was associated with an increased risk of rheumatoid arthritis; exposure to PM2.5 was associated with an increased risk of rheumatoid arthritis, connective tissue diseases (CTDs) and inflammatory bowel diseases (IBD).

**Conclusion:**

Long-term exposure to air pollution was associated with higher risk of developing autoimmune diseases, in particular rheumatoid arthritis, CTDs and IBD. Chronic exposure to levels above the threshold for human protection was associated with a 10% higher risk of developing IMIDs.

**Key messages**

**What is already known about this subject?**

- Environmental air pollution can trigger adaptive immunity.
- The association between long-term air pollution exposure and risk of autoimmune diseases is still unclear.

**What does this study add?**

- We found that long-term exposure to air pollution was associated with higher risk of autoimmune diseases.
- In particular, exposure to particulate matter (PM)10 was associated with rheumatoid arthritis, while exposure to PM2.5 was associated with rheumatoid arthritis, connective tissue diseases and inflammatory bowel diseases.

**How might this impact on clinical practice or further developments?**

- Individuals chronically exposed to high levels of air pollution might be at risk of developing autoimmune diseases.

## Introduction

Immune-mediated diseases (IMIDs) are a wide group of diseases characterised by the dysregulation and uncontrolled activation of the immune system, resulting in increased systemic inflammation and immune-mediated tissue damage.^1^ IMIDs include a broad range of rheumatic diseases such as rheumatoid arthritis, psoriatic arthritis, systemic lupus erythematosus, systemic sclerosis, connective tissue diseases (CTDs), as well as gastroenterological diseases such as inflammatory bowel diseases (IBDs) and immune-mediated neurological diseases such as multiple sclerosis. Epidemiological studies show that the incidence and the prevalence of such conditions are increasing steadily in the last decade.^2 3^ The reasons at the basis of this increasing trend are not fully known, underlining the need for a more profound understanding of pathophysiology and aetiopathogenesis of such diseases. Unrevealing the factors associated with the development of IMIDs is crucial in order to find out new and effective targets for therapies and, possibly, to set up appropriate public health prevention strategies.

Currently, it is largely accepted that the overt clinical expression of IMIDs results from the interaction between genetic predisposition and environmental factors. Air pollution is among the environmental factors that are thought to cause and exacerbate a number of IMIDs.^4^

Environmental air pollution is composed of solid particles and gaseous substances which derive predominantly from fossil fuel combustion coming from industry production and vehicle exhaust.^5^ The main components of air pollution are particulate matters (PMs), a complex mixture of chemical elements such as heavy metal, carbonaceous materials, persistent organic pollutants, volatile compounds and polycyclic aromatic hydrocarbons (PAHs), and a miscellany of gases (eg, carbon monoxide, nitrogen dioxide, ozone and sulfur dioxide).

Evidence shows that these substances can enhance expression of several inflammatory pathways, stimulate the production of cytokines and upregulate the activation of genes involved in inflammatory response.^6^ PM2.5 exposure has been associated with an increased production of interleukin (IL)-1, IL-6 and tumour necrosis factor.^7^ Furthermore, PM frequently contains microbial components, for example, lipopolysaccharides, which can activate the NLPR3 inflammasome, resulting in an increased production of proinflammatory cytokines that contribute to the local and systemic inflammation.^8^ Moreover, PMs are able to promote the generation of reactive oxygen species which, in turn, activate nuclear factor kappa B and stimulate the production of T-helper lymphocytes type 1 (Th1), resulting in DNA damage and cell death.^9^

In addition, PM exposure can negatively affect several organs and tissues, including the pulmonary, cardiovascular and central nervous systems. In the lungs, PMs can directly interact with alveolar macrophages leading to the release of Th1-type cytokines (IL-12 and interferon gamma) that generate an intense local inflammation.^10^ Oxidative stress generated by PMs can also induce dendritic cells to migrate to the local lymph nodes, cause cell necrosis and apoptosis, and contribute to the release and activation of neutrophil extracellular traps, which is accompanied by increased IL-17 and IL-23 serum levels.^11^ Moreover, local pulmonary inflammation induced by environmental air pollution participates in the pathogenesis of rheumatoid arthritis through direct citrullination of the proteins, which stimulates the secretion of anticitrullination peptide antibodies (ACPAs).^12^ Through the lungs, PMs may enter the circulation, reaching extrapulmonary tissues. Evidence suggests a link between airborne PM2.5 exposure and exacerbation of pre-existing cardiopulmonary diseases leading to increased morbidity and mortality; in addition, chronic exposure to PMs early in life is directly link to the development of significant cardiovascular alterations, including hypertension, heart failure and ischaemic heart diseases.^13 14^ Furthermore, PM2.5 is associated with higher risk of developing neurodegenerative and neurovascular conditions. PMs can cross the brain–blood barrier and reach the central nervous system, stimulating local immune responses and causing infarct of small vessels.^15^

However, despite the strong biological rationale linking air pollution exposure to the development of various autoimmune diseases, such relationship is still a matter of controversy. This debate mainly originates from the dearth of epidemiological studies on large populations investigating the association between chronic exposure to air pollution and IMIDs. The vast majority of the studies available in the literature concern rheumatoid arthritis.^16–18^ The exposure to air pollution and the proximity to streets have been associated with higher risk of rheumatoid arthritis in various epidemiological studies.^4^ Moreover, the exposure to air pollution was associated with higher chance of positivity of anticitrullinated antibodies.^19^ Recently, Park and colleagues reported a higher risk of rheumatoid arthritis but not systemic lupus erythematosus or ankylosing spondylitis in patients chronically exposed to ultrafine PM.^20^ However, another study by Bernatsky *et al* reported that exposure to ultrafine PM was associated with higher risk of immune diseases.^21^ Overall, a systematic review of eight studies published before 2016 showed that the relationship between immune-mediated rheumatic diseases and air pollution was unclear.^22^

The primary objective of the present study was to determine and describe, using a nationwide cohort, the association between long-term exposure to air pollution and IMIDs.

## Materials and methods

Clinical and demographic data were extracted from the DeFRA dataset, which originated from the homonym web-based fracture risk assessment tool, widely diffused across Italy. Data are entered in the DeFRA dataset directly by registered physicians on fracture risk calculation. DeFRA users can calculate the 10-year fracture risk in the setting of osteoporosis evaluation by entering clinical features of their patients on the website (https://defra-osteoporosi.it/), similarly to what happened with the FRAX. We retrieved data of men and women all over Italy collected from June 2016 to November 2020 by more than 3500 physicians (both family care practitioners and specialists). The DeFRA dataset contains many clinical variables such as age; weight; height; number and site of prior fragility fractures; parental history of hip and clinical vertebral fractures; glucocorticoid (GC) intake (≥5 mg/day prednisone equivalent); treatment with adjuvant hormone therapy for breast or prostate cancer; diagnosis of rheumatoid arthritis, systemic lupus erythematosus, psoriatic arthritis, systemic sclerosis, CTDs, IBDs, chronic obstructive pulmonary disease, diabetes, neurological diseases (including Parkinson’s disease, multiple sclerosis and severe physical disability); and lumbar spine and femoral neck T-scores (calculated from the bone mineral density (BMD) reference range of young same-sex individuals). The dataset characteristics have been described in detail elsewhere^23–26^; in this paper, the dataset has been updated to November 2020. The DeFRA dataset has been used for similar purposes of the present study in another recently published paper.^25^

Data on the air pollution (PM10 and PM2.5 concentrations) were provided by the Italian Institute of Environment Protection and Research, which collects daily data from air quality stations across Italy. The long-term average PM concentrations were the exposure of interest. Every study subject was linked to a PM exposure value, which resulted from the average concentration of urban, rural and near-traffic stations of the subject province of residency from January 2013 to November 2020. Patients were linked to the nearest air quality station through zip code centroids.

Group comparisons were performed using the Student t-test and the Mann-Whitney U test (for normally and non-normally distributed continuous variables, respectively). Associations between continuous variables were tested using Pearson correlation coefficients. Generalised linear models with robust estimators were employed to identify determinants of IMIDs. Exposure to PMs was analysed either as a continuous variable or as a binary variable (exposure thresholds were 30 µg/m^3^ for PM10 and 20 µg/m^3^ PM2.5). We approached the analysis treating PM exposure both as a continuous variable and a dichotomous variable. We did so because we presumed that there might be a threshold effect of exposure to PM. Such effect might be blunted or reduced in intensity when treating the PM exposure as a continuous variable and should be more apparent when considering thresholds. We chose the thresholds that are generally considered as harmful for human health. The fully adjusted model included age, body mass index, menopause, glucocorticoid treatment, treatment with adjuvant hormone therapy for breast or prostate cancer, specialty of the physician who entered the data and macroarea of residency (stratified as a categorical variable: northern Italy, central Italy and southern Italy). Differences were considered significant at a p value of <0.05. All statistical analyses were performed using SPSS V.26. Data were anonymised in full compliance with the Italian Code of Protection of Personal Data (Legislative Decree 196/03, http:// www.camera.it/parlam/leggi/deleghe/03196dl.htm). No identifiers related to patients were provided to the researchers. Results derived from all analyses were produced as aggregated summaries, which are not possible to assign, either directly or indirectly, to the individual patients.

## Results

**Figure 1:**
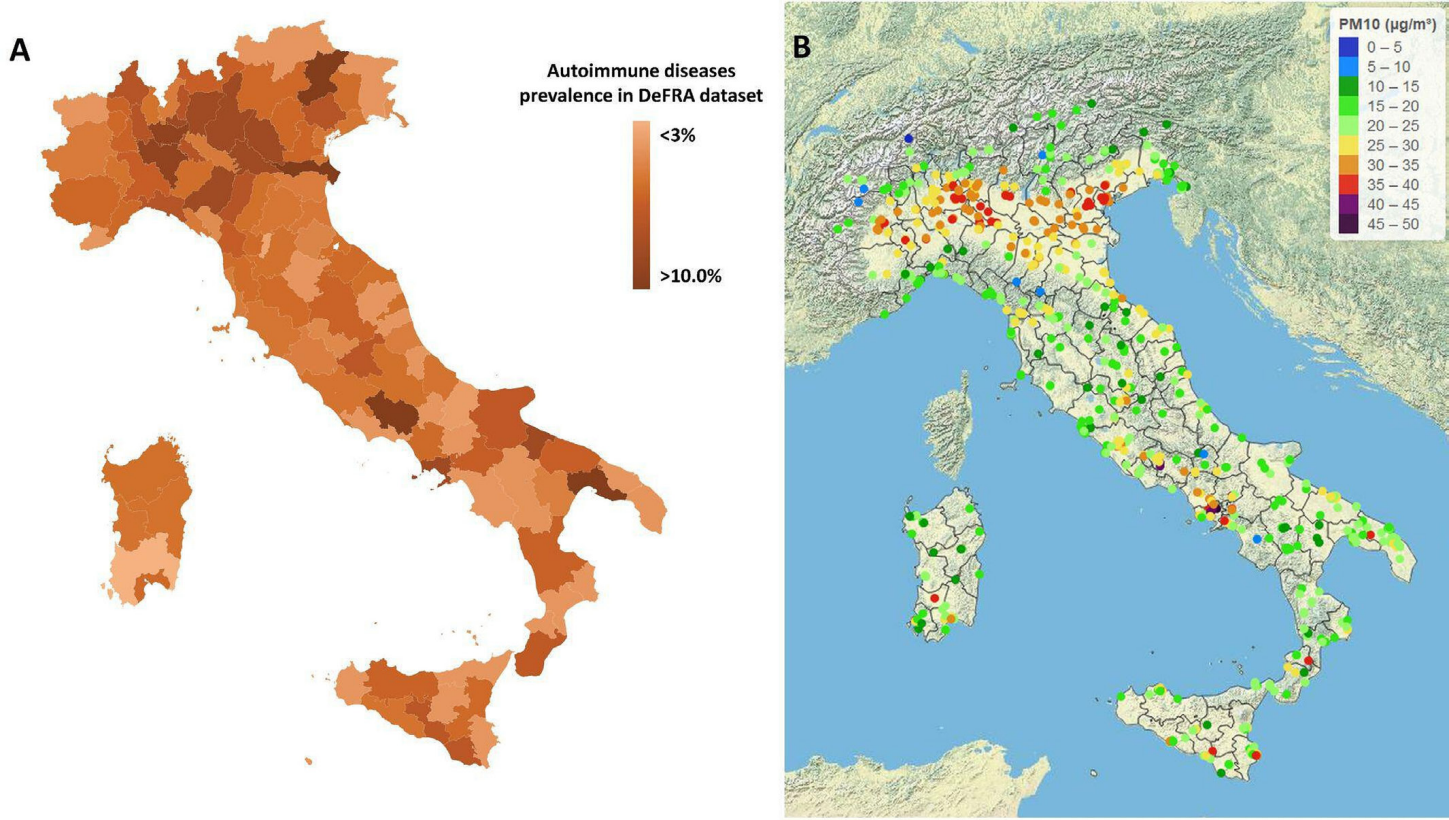
(A) Prevalence of autoimmune diseases across Italy in the DeFRA database. (B) Long-term exposure to PM of less than 10 µm in Italy (2013–2019 average concentration in μg/m^3^). PM, particulate matter.

**Figure 2:**
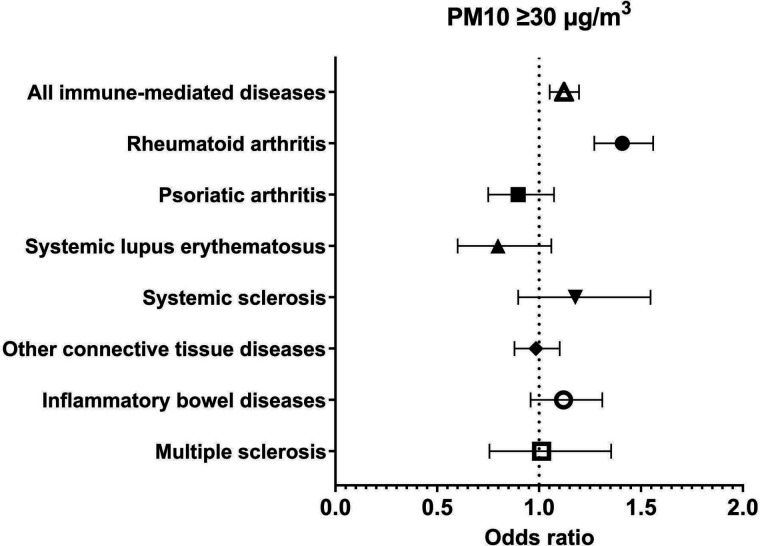
Risk of immune-mediated conditions at chronic exposure to PM10 ≥30 µg/m^3^. PM, particulate matter.

**Figure 3:**
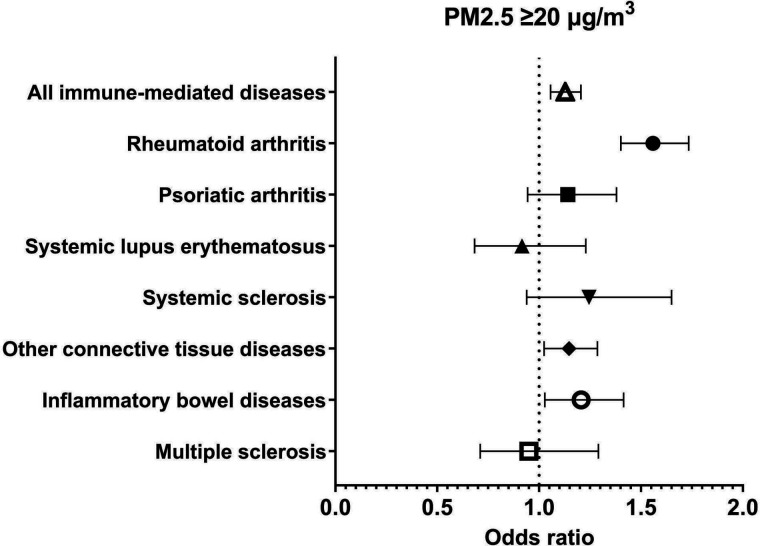
Risk of immune-mediated conditions at chronic exposure to PM2.5≥20 µg/m^3^. PM, particulate matter.

A total of 81 363 subjects were enrolled in the study. The vast majority were female (91.9%) and 17 866 (22%) presented at least one comorbidity. Among these, 9723 (11.9% of the whole cohort) were diagnosed with an autoimmune disease. Table 1 shows the general characteristics of the cohort. Data on air quality were obtained from 617 air quality stations across 110 Italian provinces. Average long-term exposure in Italy (period of data collection 2013–2019) was 16.0 µg/m^3^ for PM2.5 and 25.0 µg/m^3^ for PM10 (figure 1). Exposure to air pollution was above average in northern Italy, Po Valley and other near-city areas. We found a positive, yet small, association between exposure to PM10 and the risk of being diagnosed with an autoimmune disease (ρ 0.007, p 0.014; fitting of the fully adjusted model was good with deviance/df of 0.788). This finding translates into a 7% higher risk of having any autoimmune disease every 10 µg/m^3^ increase in PM10 concentration. As regards PM2.5, we did not find any association with the risk of autoimmune diseases (ρ 0.001, p 0.751). Table 2 shows the results of the generalised linear model for each immune-mediated condition considered separately. We then analysed the exposure to air pollution as categorical variables (threshold of chronic exposure was set at 30 µg/m^3^ for PM10 and 20 µg/m^3^ for PM2.5). In the adjusted binary logistic regression model, we found that subjects chronically exposed to levels of PM10 above 30 µg/m^3^ had a 13% higher risk of having any autoimmune disease (adjusted OR (aOR) 1.122, 95% CI 1.052 to 1.196). As regards chronic exposure to PM2.5, we found that patients exposed to levels higher than 20 µg/m^3^ had a 13% higher risk of developing autoimmune diseases (aOR 1.128, 95% CI 1.056 to 1.205). Figures 2 and 3 show the effects of chronic exposure above the mentioned threshold of PMs on the risk of autoimmune diseases. Exposure to high levels of PM10 were associated with an increased risk of rheumatoid arthritis (aOR 1.408, 95% CI 1.271 to 1.560) but no other autoimmune diseases, whereas exposure to high levels of PM2.5 was associated with an increased risk of rheumatoid arthritis (aOR 1.559, 95% CI 1.401 to 1.734), CTDs (aOR 1.147, 95% CI 1.024 to 1.286) and IBDs (1.206, 95% CI 1.028 to 1.415) but no other autoimmune diseases.

**Table 1:** General characteristics of the cohort

Overall cohort		N=81 363
Age (years) (±SD)		65 (11)
Sex, female (%)		74 772 (91.9)
Menopause, n (% of female)		71 654 (95.8)
Weight (±SD)		62.2 (12.0)
Height (±SD)		160 (8)
Concomitant treatment (%)	None	69 616 (85.6)
	Treatment with adjuvant hormone therapy	6264 (7.7)
	Glucocorticoids ≥5 mg/day of prednisone equivalent for ≥3 months	5483 (6.7)
Diseases (%)	None	63 497 (78.0)
	Rheumatoid arthritis	3817 (4.7)
	Psoriatic arthritis	992 (1.2)
	Systemic lupus erythematosus	384 (0.5)
	Systemic sclerosis	374 (0.5)
	Other connective tissue diseases	2571 (3.2)
	Inflammatory bowel diseases	1250 (1.5)
	Multiple sclerosis	335 (0.4)
	Diabetes	4184 (5.1)
	Chronic pulmonary diseases	2097 (2.6)
	Severe physical handicap	1058 (1.3)
	Parkinson’s disease	502 (0.6)
	HIV infection	302 (0.4)
Specialty of the physician entering patients’ data (%)	Rheumatology	17 310 (21.3)
	Endocrinology	26 278 (32.3)
	Family care practitioner	10 207 (12.5)
	Internal medicine	5757 (7.1)
	Orthopedy	5699 (7.0)
	Physiatry	5420 (6.7)
	Gynaecologist	2328 (2.9)
	Other	8364 (10.3)

**Table 2:** Association between air pollution exposure and risk of autoimmune diseases

Diseases	PM10, β (95% CI)	P value	PM2.5, β (95% CI)	P value
All autoimmune diseases	0.007 (0.001 to 0.013)	**0.014**	0.001 (−0.006 to 0.008)	NS
Rheumatoid arthritis	0.005 (−0.004 to 0.014)	NS	0.022 (0.011 to 0.034)	**0.0001**
Psoriatic arthritis	0.003 (−0.002 to 0.005)	NS	0.004 (−0.016 to 0.023)	NS
Systemic lupus erythematosus	−0.007 (−0.033 to 0.019)	NS	−0.014 (−0.046 to 0.017)	NS
Systemic sclerosis	0.013 (−0.011 to 0.038)	NS	0.013 (−0.017 to 0.042)	NS
Other connective tissue diseases	−0.004 (−0.014 to 0.007)	NS	0.015 (0.003 to 0.028	**0.013**
Multiple sclerosis	0.025 (−0.001 to 0.051)	**0.063**	0.036 (0.006 to 0.067)	**0.019**

NS, not significant; PM, particulate matter.

## Discussion

Herein we presented an observational, nationwide cohort study on the association between long-term exposure to fine PM and prevalence of autoimmune diseases. We found that exposure to air pollution was associated with higher prevalence of IMIDs. In particular, we witnessed an association with rheumatoid arthritis, CTDs and IBDs in patients exposed to high levels of PM2.5.

The biological rationale supporting our results is strong. Our study has been inspired by numerous preclinical studies and several clinical observational studies. Exposure to air pollution has been linked with dysregulation of the adaptive immune system with impairment of host response and abnormal cytokine secretion.^7^ As an example, exposure to PAHs, assessed at individual level by measuring urinary 1-hydroxypyrene, has been associated with decreased number of circulating regulatory T cells and lower levels of IL-10.^27^ This detrimental effect on the immune system has been proved to be mediated by the oxidative stress induced by air pollution, which is a well-known proinflammatory stimulus.^28^ Numerous studies have also demonstrated that cigarette smoking can trigger adaptive immunity and lead to the development of autoimmune diseases. A paradigmatic example comes from rheumatoid arthritis, in which cigarette smoking represents a major predisposing factor.^29^ Indeed, smoking habits has been associated with a twofold higher risk of seropositive rheumatoid arthritis.^30^ Smoke can directly elicit the post-translational citrullination of proteins in the lower and upper airways; these citrullinated proteins are, in turn, responsible of the production of ACPA.^12^ Remarkably, cigarette smoking shares various toxics with emission from fossil fuels. As a matter of fact, diesel exhaust has been shown to induce peptidyl arginine deiminase activity in the bronchial epithelium.^31^ These preclinical findings have been supported by several, real-life, clinical observations as well. Indeed, air pollution exposure has been associated with an increased risk of developing rheumatoid arthritis^16^ and, more recently, with a higher risk of experience flares.^32 33^ Moreover, the exposure to traffic-derived pollutants has been linked with poorer response to biological treatments in chronic inflammatory arthritides.^34 35^ With our nationwide experience, we further confirmed such results. We found that the prevalence of rheumatoid arthritis was nearly 50% higher in subjects exposed to high levels of PMs. This finding is line with a meta-analysis of previously published studies on the topic.^17^ Interestingly, the authors of the meta-analysis found that PM2.5 appeared to confer a higher risk of rheumatoid arthritis as compared with PM10 or other toxics. The incidence and prevalence of IBDs are increasing worldwide, with accelerating incidence in highly industrialised countries.^36^ Remarkably, only the exposure to gaseous pollutants and ultrafine PMs and not coarse PMs were associated with an increased risk of IBDs,^37^ a result that is somehow in line with our findings. Indeed, we found that exposure to PM2.5 but not PM10 was associated with a significant increase in the prevalence IBDs. The incidence of CTDs is increasing worldwide, and the larger increase has been witnessed in western and industrialised countries,^38^ a result that is, again, consistent with our findings.

Of note, PM2.5 molecules, given their small diameter, are less affected by rain and weather conditions compared with larger PMs.^39^ Indeed, PM2.5 concentrations tend to do not fluctuate in response to rain and might represent a more accurate proxy for chronic exposure to air pollution than PM10. This evidence might explain the discrepancy that we found in our analysis.

We found that the prevalence of some immune-mediated conditions was higher in patients exposed to fine PM. However, we did not find any significant association between psoriatic arthritis, systemic sclerosis, systemic lupus erythematosus or multiple sclerosis and exposure to air pollutants. To date, there is uncertainty regarding the relationship between such diseases and exposure to air pollution. As an example, there are no clinical studies linking air pollution and psoriatic arthritis, despite the robust association between cigarette smoking and psoriasis that has been demonstrated,^40^ and a few studies have shown a possible detrimental effect of air pollution on psoriasis extension.^41^ Another example comes for systemic sclerosis. Air pollution exposure has been linked with higher burden of Raynaud phenomenon and pulmonary manifestations,^42^ but no solid evidence has been produced regarding the association between exposure to traffic-derived pollutants and the risk of developing systemic sclerosis. As regards systemic lupus erythematosus, the clinical data are still controversial, but there is some evidence that air pollution is associated with this disease.^43^ In contrast, we did not find any association between systemic lupus erythematosus and PM exposure, but the number of patients at risk was too small to draw strong conclusions. Finally, many studies have been published on the association between multiple sclerosis and air pollution. However, these studies yielded controversial results, with some indicating an association^44^ and others rejecting this hypothesis.^45^ In our analysis, we found that multiple sclerosis was significantly associated with air pollution exposure only when the PM exposure was treated as a continuous variable.

In our analysis, we found different results when considering PM exposure as a continuous variable or as a dichotomous variable. This seemingly controversial result might be related to a sort of deterministic effect of PM exposure on the risk of autoimmune diseases. Deterministic phenomena are usually characterised by thresholds below which the outcome does not occur. In contrast, stochastic effects are characterised by increasing chances of the effects as the exposure increases. In this scenario, we might hypothesise that treating the exposure as a continuous variable might have, somehow, diluted the effect.

There are three types of approaches to measure the exposure to environmental air pollution. The first (the one that we used) is the air monitoring through air quality stations; the second is direct exposure through personal monitors; and the third requires the measurement of biological markers that are related to exposure (eg, chemicals directly measured in the blood). All these three methods have shortcomings and advantages. Air monitoring might be inaccurate in assessing personal exposure, yet it allows an estimation of the long-term exposure. Personal monitor represents the more precise method but it is limited by the ‘Hawthorne effect’ (ie, changing behaviour of subjects in study related to increased awareness). Finally, the measurement of biological metabolites of air pollutants is costly and, in most of the cases, it can only examine the acute exposure to pollutants. To the best of our knowledge, all the epidemiological studies that linked immune-mediated diseases with air pollution were conducted using data from air monitoring stations or similar approaches (eg, proximity to street).^46^

Our study should be interpreted in light of some strengths and limitations. The key strength of our study is the large sample size and access to the diagnosis of many IMIDs. Moreover, the cohort was evenly distributed across provinces that largely differed on PM exposure. Although our results are not generalisable to the entire Italian population, our sample is highly representative of the female population aged 50 years or more. However, since the dataset collects prevalently data of women at high risk of fracture, the prevalence of autoimmune diseases might not reflect the real prevalence in the general population of such diseases, possibly affecting the generalisability of our results. Moreover, the date of the diagnosis and onset of autoimmune diseases were not available; hence, we cannot rule out with certainty a possible reverse causality bias. The chronic exposure pollution has been estimated from the mean concentration of PMs between 2013 and 2019. The exposure of interest might be imprecise and might just represent a rough estimation of the long-term exposure. However, this method is the simplest available and has been largely used in the literature for similar purposes.^47^ Indeed, we assumed that the variation of PM was constant within Italy in the decades before the measurement. We cannot rule out local variations of PM exposure prior to the period of 2013–2020 due to expanding cities or industrial centres. However, we did not have access to pollution models for estimating historic concentrations that covered the age of the study population. In addition, we did not have access to scholarity, climate, socioeconomic status, smoking status and other relevant covariates such as occupational factors that can affect the prevalence of IMIDs. Nevertheless, the large sample size and adjustment for macroarea of residency should have attenuated such confounding. Moreover, smoking and socioeconomic status might not represent major confounders; indeed, it has been demonstrated that, conversely to what was expected, regions with higher concentrations of PM had higher income and less smokers.^48^ This inverse association is particularly true in Italy, where the Po Valley represents the most polluted area of Italy by far but has the lowest prevalence of smokers and the highest gross domestic product.^49^ Thus, smoking and socioeconomical status might only attenuate our results, without affecting the overall validity. Nevertheless, the lack of information regarding socioeconomic factors and smoking represents a major limitation of the study. Indeed, patients with higher scholarity and income might be more prone avoiding highly polluted areas and might be more likely to seek care for their autoimmune diseases, possibly introducing selection bias. Furthermore, we did not have access to other types of pollutants, which might be also associated with higher risk of immune diseases, introducing a possible confounder. However, water and soil pollutants are not collected extensively, making it difficult to reduce the impact of such confounding. In addition, we should acknowledge some limitations common to all large datasets that originated from physician-reported data. For example, misclassifications and incorrect diagnosis might have affected our findings. Moreover, the dataset was originally designed to study determinants of fracture. The way the data are recorded may introduce a recruitment bias, or some characteristics of these patients (who are essentially women) may confound the observed association. Specifically, the dataset over-represents postmenopausal women, who are also more prone to some autoimmune diseases. In this regard, systemic lupus erythematosus, which generally affects younger women, does not appear to be increased by air pollution, but, by design, there are too few exposed young women in this dataset, especially for a relatively rare disease. In addition, we did not have information regarding the seropositivity of rheumatoid arthritis patients. Indeed, noxious airway agents have been associated with the production of ACPA and seropositive rheumatoid arthritis.

In conclusion, we found that the exposure to fine PMs was associated with an increased risk of some autoimmune diseases. Chronic exposure to traffic and industrial derived pollutants was associated with approximately 40% higher risk of rheumatoid arthritis, 20% higher risk of IBDs and 15% higher risk of CTDs.

## Data Availability

Data are available upon reasonable request.

## References

[1] Rose NR. Prediction and prevention of autoimmune disease in the 21st century: a review and preview. Am J Epidemiol 2016;183:403–6. 10.1093/aje/kwv292 26888748

[2] Cross M, Smith E, Hoy D, et al. The global burden of rheumatoid arthritis: estimates from the global burden of disease 2010 study. Ann Rheum Dis 2014;73:1316–22. 10.1136/annrheumdis-2013-204627 24550173

[3] Dinse GE, Parks CG, Weinberg CR, et al. Increasing prevalence of antinuclear antibodies in the United States. Arthritis Rheumatol 2020;72:1026–35. 10.1002/art.41214 32266792 PMC7255943

[4] Zhao C-N, Xu Z, Wu G-C, et al. Emerging role of air pollution in autoimmune diseases. Autoimmun Rev 2019;18:607–14. 10.1016/j.autrev.2018.12.010 30959217

[5] Ambient (outdoor) air pollution. Available: https://www.who.int/news-room/fact-sheets/detail/ambient-(outdoor)-air-quality-and-health

[6] Gawda A, Majka G, Nowak B, et al. Air pollution, oxidative stress, and exacerbation of autoimmune diseases. Cent Eur J Immunol 2017;42:305–12. 10.5114/ceji.2017.70975 29204097 PMC5708213

[7] Chen R, Li H, Cai J, et al. Fine particulate air pollution and the expression of microRNAs and circulating cytokines relevant to inflammation, coagulation, and vasoconstriction. Environ Health Perspect 2018;126:017007. 10.1289/EHP1447 29342453 PMC6014692

[8] Uh S-T, Koo SM, Kim Y, et al. The activation of NLRP3-inflammsome by stimulation of diesel exhaust particles in lung tissues from emphysema model and RAW 264.7 cell line. Korean J Intern Med 2017;32:865–74. 10.3904/kjim.2016.033 28814068 PMC5583452

[9] Morgan MJ, Liu Z-gang, Liu Z. Crosstalk of reactive oxygen species and NF-κB signaling. Cell Res 2011;21:103–15. 10.1038/cr.2010.178 21187859 PMC3193400

[10] Park E-J, Roh J, Kim Y, et al. Pm 2.5 collected in a residential area induced Th1-type inflammatory responses with oxidative stress in mice. Environ Res 2011;111:348–55. 10.1016/j.envres.2010.11.001 21256479

[11] Stark MA, Huo Y, Burcin TL, et al. Phagocytosis of apoptotic neutrophils regulates granulopoiesis via IL-23 and IL-17. Immunity 2005;22:285–94. 10.1016/j.immuni.2005.01.011 15780986

[12] Valesini G, Gerardi MC, Iannuccelli C, et al. Citrullination and autoimmunity. Autoimmun Rev 2015;14:490–7. 10.1016/j.autrev.2015.01.013 25636595

[13] Yang S, Lee S-P, Park J-B, et al. PM2.5 concentration in the ambient air is a risk factor for the development of high-risk coronary plaques. Eur Heart J Cardiovasc Imaging 2019;20:1355–64. 10.1093/ehjci/jez209 31410457

[14] Calderón-Garcidueñas L, Vincent R, Mora-Tiscareño A, et al. Elevated plasma endothelin-1 and pulmonary arterial pressure in children exposed to air pollution. Environ Health Perspect 2007;115:1248–53. 10.1289/ehp.9641 17687455 PMC1940106

[15] Calderón-Garcidueñas L, Solt AC, Henríquez-Roldán C, et al. Long-Term air pollution exposure is associated with neuroinflammation, an altered innate immune response, disruption of the blood-brain barrier, ultrafine particulate deposition, and accumulation of amyloid beta-42 and alpha-synuclein in children and young adults. Toxicol Pathol 2008;36:289–310. 10.1177/0192623307313011 18349428

[16] Hart JE, Källberg H, Laden F, et al. Ambient air pollution exposures and risk of rheumatoid arthritis: results from the Swedish EIRA case-control study. Ann Rheum Dis 2013;72:888–94. 10.1136/annrheumdis-2012-201587 22833374 PMC3654032

[17] Di D, Zhang L, Wu X, et al. Long-Term exposure to outdoor air pollution and the risk of development of rheumatoid arthritis: a systematic review and meta-analysis. Semin Arthritis Rheum 2020;50:266–75. 10.1016/j.semarthrit.2019.10.005 31761290

[18] Hart JE, Källberg H, Laden F, et al. Ambient air pollution exposures and risk of rheumatoid arthritis. Arthritis Care Res 2013;65:1190–6. 10.1002/acr.21975 PMC3659202 23401426

[19] Bernatsky S, Smargiassi A, Joseph L, et al. Industrial air emissions, and proximity to major industrial emitters, are associated with anti-citrullinated protein antibodies. Environ Res 2017;157:60–3. 10.1016/j.envres.2017.04.035 28525857

[20] Park JS, Choi S, Kim K, et al. Association of particulate matter with autoimmune rheumatic diseases among adults in South Korea. Rheumatology 2021;60:5117–26. 10.1093/rheumatology/keab127 33560298 PMC8566218

[21] Bernatsky S, Smargiassi A, Barnabe C, et al. Fine particulate air pollution and systemic autoimmune rheumatic disease in two Canadian provinces. Environ Res 2016;146:85–91. 10.1016/j.envres.2015.12.021 26724462

[22] Sun G, Hazlewood G, Bernatsky S, et al. Association between air pollution and the development of rheumatic disease: a systematic review. Int J Rheumatol 2016;2016:1–11. 10.1155/2016/5356307 PMC5099457 27847517

[23] Adami G, Giollo A, Rossini M, et al. Different fracture risk profile in patients treated with anti-osteoporotic drugs in real-life. Reumatismo 2020;72:71–4. 10.4081/reumatismo.2020.1267 32700872

[24] Adami G, Gatti D, Rossini M, et al. Factors associated with referral for osteoporosis care in men: a real-life study of a nationwide dataset. Arch Osteoporos 2021;16:56. 10.1007/s11657-021-00915-8 33723677

[25] Adami G, Cattani G, Rossini M, et al. Association between exposure to fine particulate matter and osteoporosis: a population-based cohort study. Osteoporos Int 2022;33:169-176. 10.1007/s00198-021-06060-9 34268604 PMC8758604

[26] Adami G, Gatti D, Rossini M, et al. Risk of fragility fractures in obesity and diabetes: a retrospective analysis on a nation-wide cohort. Osteoporos Int 2020;31:2113–22. 10.1007/s00198-020-05519-5 32613408

[27] Yao Y, Wang D, Ma H, et al. The impact on T-regulatory cell related immune responses in rural women exposed to polycyclic aromatic hydrocarbons (PAHs) in household air pollution in Gansu, China: a pilot investigation. Environ Res 2019;173:306–17. 10.1016/j.envres.2019.03.053 30951957

[28] Li Z, Liu Q, Xu Z, et al. Association between short-term exposure to ambient particulate air pollution and biomarkers of oxidative stress: a meta-analysis. Environ Res 2020;191:110105. 10.1016/j.envres.2020.110105 32835677

[29] Prisco LC, Martin LW, Sparks JA. Inhalants other than personal cigarette smoking and risk for developing rheumatoid arthritis. Curr Opin Rheumatol 2020;32:279–88. 10.1097/BOR.0000000000000705 32141952 PMC7233294

[30] Ishikawa Y, Terao C. The impact of cigarette smoking on risk of rheumatoid arthritis: a narrative review. Cells 2020;9:475. 10.3390/cells9020475 PMC7072747 32092988

[31] Colasanti T, Fiorito S, Alessandri C, et al. Diesel exhaust particles induce autophagy and citrullination in normal human bronchial epithelial cells. Cell Death Dis 2018;9:1073. 10.1038/s41419-018-1111-y 30341285 PMC6195610

[32] Adami G, Viapiana O, Rossini M, et al. Association between environmental air pollution and rheumatoid arthritis flares. Rheumatology 2021;60:4591-4597. 10.1093/rheumatology/keab049 33470401

[33] Ingegnoli F, Ubiali T, Schioppo T, et al. Potential short-term air pollution effects on rheumatoid arthritis activity in metropolitan areas in the North of Italy: a cross-sectional study. Int J Environ Res Public Health 2021;18:8490. 10.3390/ijerph18168490 34444236 PMC8392349

[34] Adami G, Rossini M, Viapiana O, et al. Environmental air pollution is a predictor of poor response to biological drugs in chronic inflammatory arthritides. ACR Open Rheumatol 2021;3:451–6. 10.1002/acr2.11270 34060251 PMC8280800

[35] Zhao N, Bernatsky S. Is air pollution linked with poor response to biologics? Nat Rev Rheumatol 2021;17:583–4. 10.1038/s41584-021-00681-4 34349255

[36] Ng SC, Shi HY, Hamidi N, et al. Worldwide incidence and prevalence of inflammatory bowel disease in the 21st century: a systematic review of population-based studies. Lancet 2017;390:2769–78. 10.1016/S0140-6736(17)32448-0 29050646

[37] Kaplan GG, Hubbard J, Korzenik J, et al. The inflammatory bowel diseases and ambient air pollution: a novel association. Am J Gastroenterol 2010;105:2412–9. 10.1038/ajg.2010.252 20588264 PMC3180712

[38] Smith E, Hoy DG, Cross M, et al. The global burden of other musculoskeletal disorders: estimates from the global burden of disease 2010 study. Ann Rheum Dis 2014;73:1462–9. 10.1136/annrheumdis-2013-204680 24590181

[39] Borge R, Requia WJ, Yagüe C, et al. Impact of weather changes on air quality and related mortality in Spain over a 25 year period [1993-2017]. Environ Int 2019;133:105272. 10.1016/j.envint.2019.105272 31675571

[40] Armstrong AW, Harskamp CT, Dhillon JS, et al. Psoriasis and smoking: a systematic review and meta-analysis. Br J Dermatol 2014;170:304–14. 10.1111/bjd.12670 24117435

[41] Abolhasani R, Araghi F, Tabary M, et al. The impact of air pollution on skin and related disorders: a comprehensive review. Dermatol Ther 2021;34:e14840. 10.1111/dth.14840 33527709

[42] Schioppo T, De Lucia O, Murgo A, et al. The burden of air pollution and temperature on Raynaud's phenomenon secondary to systemic sclerosis. Epidemiol Prev 2020;44:218–27. 10.19191/EP20.4.P228.052 32921028

[43] Jung C-R, Chung W-T, Chen W-T, et al. Long-term exposure to traffic-related air pollution and systemic lupus erythematosus in Taiwan: a cohort study. Sci Total Environ 2019;668:342–9. 10.1016/j.scitotenv.2019.03.018 30852211

[44] Bergamaschi R, Monti MC, Trivelli L, et al. PM_2.5_ exposure as a risk factor for multiple sclerosis. An ecological study with a Bayesian mapping approach. Environ Sci Pollut Res Int 2021;28:2804–9. 10.1007/s11356-020-10595-5 32894443 PMC7788018

[45] Bai L, Burnett RT, Kwong JC, et al. Long-term exposure to air pollution and the incidence of multiple sclerosis: a population-based cohort study. Environ Res 2018;166:437–43. 10.1016/j.envres.2018.06.003 29940476

[46] Sigaux J, Biton J, André E, et al. Air pollution as a determinant of rheumatoid arthritis. Joint Bone Spine 2019;86:37–42. 10.1016/j.jbspin.2018.03.001 29524589

[47] Han B, Hu L-W, Bai Z. Human exposure assessment for air pollution. Adv Exp Med Biol 2017;1017:27–57. 10.1007/978-981-10-5657-4_3 29177958

[48] Villeneuve PJ, Goldberg MS, Burnett RT, et al. Associations between cigarette smoking, obesity, sociodemographic characteristics and remote-sensing-derived estimates of ambient PM2.5: results from a Canadian population-based survey. Occup Environ Med 2011;68:920–7. 10.1136/oem.2010.062521 21610265

[49] Gallus S, Pacifici R, Colombo P, et al. Prevalence of smoking and attitude towards smoking regulation in Italy, 2004. Eur J Cancer Prev 2006;15:77–81. 10.1097/01.cej.0000180667.89087.b9 16374235

